# Supplementary material for: Proteogenomic landscape and clinical characterization of GH-producing pituitary adenomas/somatotroph pituitary neuroendocrine tumors
Source: Commun Biol. 2022 Nov 27;5:1304. doi: 10.1038/s42003-022-04272-1 (PMC9701206; doi:10.1038/s42003-022-04272-1)
Supplement: Supplementary file 2 — Supplementary Material [file 42003_2022_4272_MOESM2_ESM.pdf]

Supplementary material

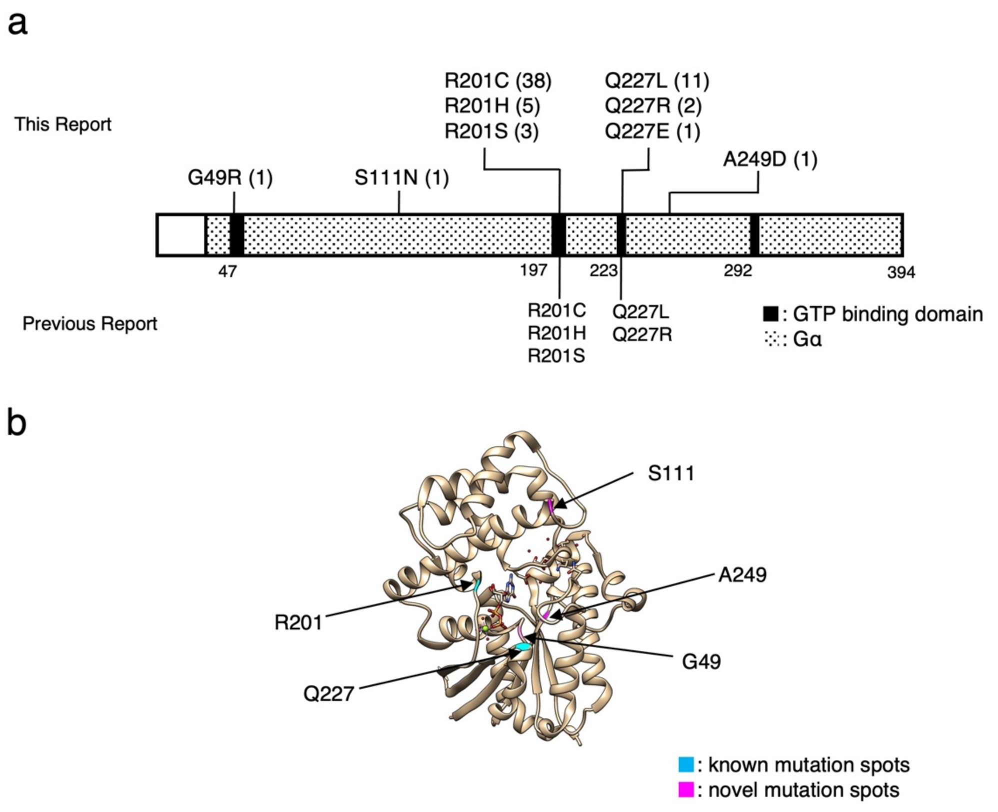

Supplementary Figure 1. (a) Two-dimensional structure of the Gsα protein. *GNAS* mutations detected in our cohort are described at the top of the schema. Mutational hotspots detected in previous reports are described at the bottom of the schema. (b) Crystal structure of the Gsα protein (PDB: 6AU6). Blue-labeled amino acids indicate known *GNAS* mutations, whereas magenta amino acids indicate novel mutations identified in the present study.

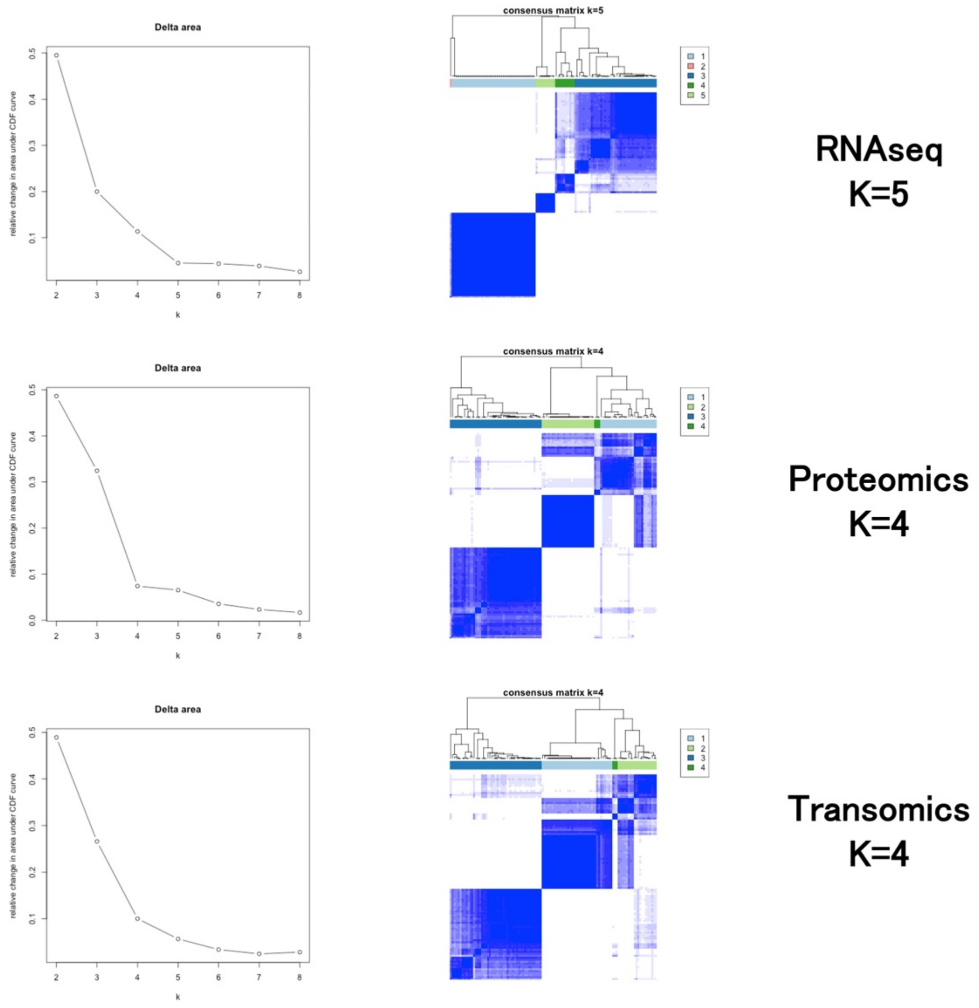

Supplementary Figure 2. Graph showing cluster metrics (y-axis) as a function of the number of cluster K (x-axis). RNA sequence, proteomics, and multiomics results are shown.

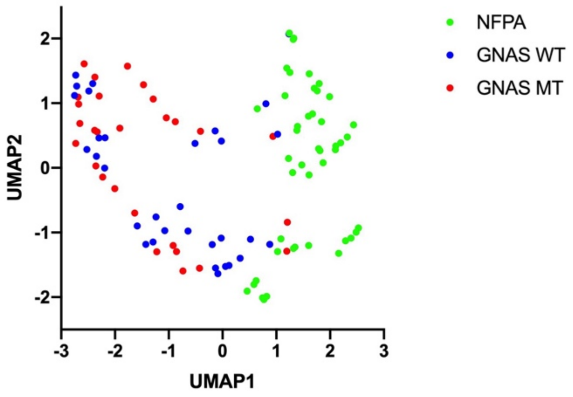

Supplementary Figure 3. Uniform manifold approximation and projection (UMAP) analysis showing clusters of pituitary adenomas according to endocrinological function.

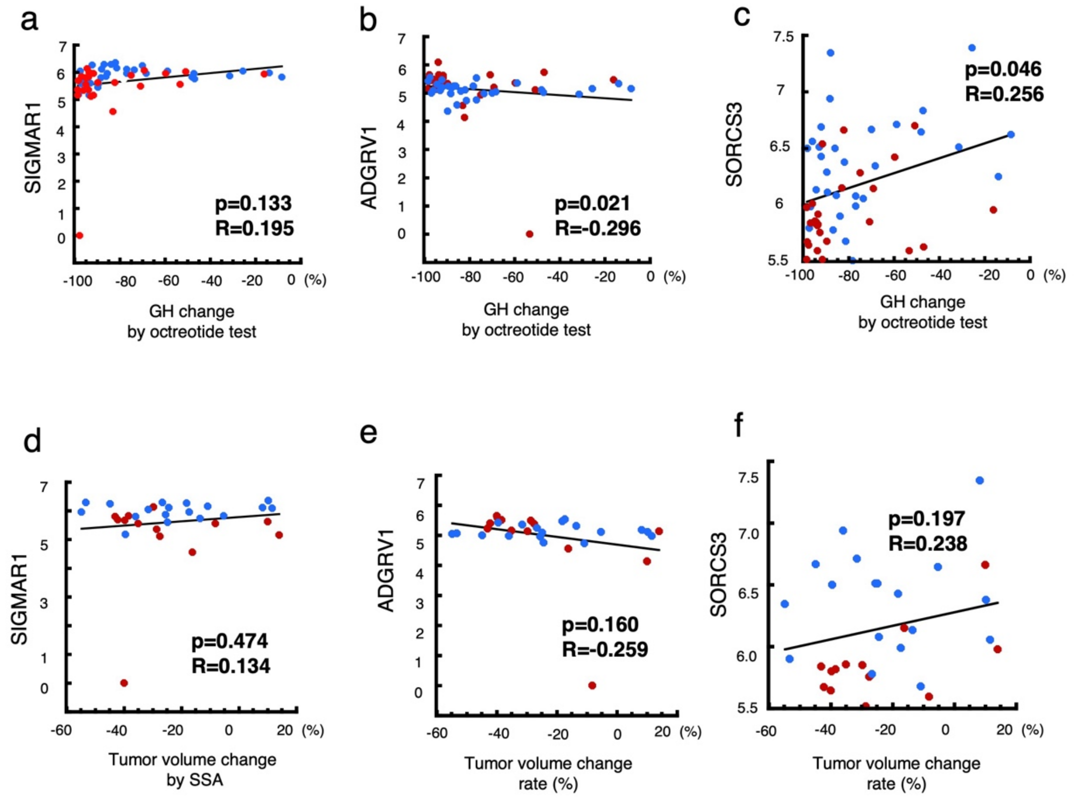

Supplementary Figure 4. Correlations between (a) sigma nonopioid intracellular receptor-1 (SIGMAR1), (b) adhesion G protein–coupled receptor V1 (ADGRV1), and (c) sortilin-related VPS10 domain–containing receptor 3 (SORCS3) protein expression level and the growth hormone (GH) change rate in the octreotide loading test (%). Correlations between (d) SIGMAR1, (e) ADGRV1, and (f) SORCS3 protein expression levels and the tumor volume change rate in the somatostatin analog (SSA) test (%). The protein expression value was log (base 10) transformed. Data were analyzed by Pearson's correlation analysis.

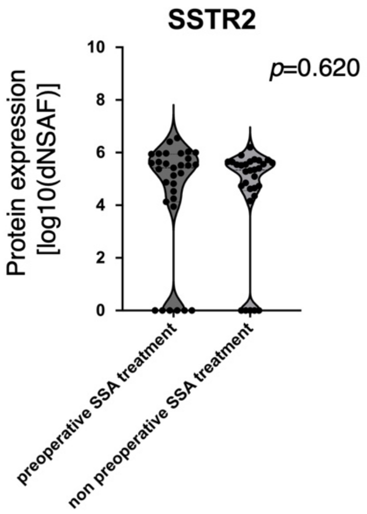

Supplementary Figure 5. Protein expression levels of SSTR2 in GH-producing pituitary adenomas/somatotroph PitNETs with or without preoperative SSA treatment.
