# Supplementary material for: Proteogenomic landscape and clinical characterization of GH-producing pituitary adenomas/somatotroph pituitary neuroendocrine tumors
Source: Commun Biol. 2022 Nov 27;5:1304. doi: 10.1038/s42003-022-04272-1 (PMC9701206; doi:10.1038/s42003-022-04272-1)
Supplement: Supplementary file 3 — Description of Additional Supplementary Files [file 42003_2022_4272_MOESM3_ESM.pdf]

Description of Additional Supplementary Files

File name: Supplementary Data 1

Description: Clinical and genetics information of all acromegaly patients

File name: Supplementary Data 2

Description: Genes analyzed by target capture sequence

File name: Supplementary Data 3

Description: Clinical information of all patients with non-functional pituitary adenoma

File name: Supplementary Data 4

Description: KEGG analysis of molecular groups that showed significantly higher expression fluctuations in cluster 3 compared to cluster 1 in consensus clustering-based proteomics

File name: Supplementary Data 5

Description: Gene ontology terms of molecular groups that showed significant expression fluctuations with and without GNAS mutations in proteomics

File name: Supplementary Data 6

Description: The source data behind the graphs in the paper
