# Supplementary material for: Proteogenomic landscape and clinical characterization of GH-producing pituitary adenomas/somatotroph pituitary neuroendocrine tumors
Source: Commun Biol. 2022 Nov 27;5:1304. doi: 10.1038/s42003-022-04272-1 (PMC9701206; doi:10.1038/s42003-022-04272-1)
Supplement: Supplementary file 10 — Reporting Summary [file 42003_2022_4272_MOESM10_ESM.pdf]

nature portfolio

Corresponding author(s): Tomoaki Tanaka

Last updated by author(s): Nov 8, 2022

Reporting Summary

Nature Portfolio wishes to improve the reproducibility of the work that we publish. This form provides structure for consistency and transparency in reporting. For further information on Nature Portfolio policies, see our Editorial Policies and the Editorial Policy Checklist.

Statistics

For all statistical analyses, confirm that the following items are present in the figure legend, table legend, main text, or Methods section.

n/a	Confirmed	
☐	☒	The exact sample size (*n*) for each experimental group/condition, given as a discrete number and unit of measurement
☐	☒	A statement on whether measurements were taken from distinct samples or whether the same sample was measured repeatedly
☐	☒	The statistical test(s) used AND whether they are one- or two-sided<br>*Only common tests should be described solely by name; describe more complex techniques in the Methods section.*
☐	☒	A description of all covariates tested
☐	☒	A description of any assumptions or corrections, such as tests of normality and adjustment for multiple comparisons
☐	☒	A full description of the statistical parameters including central tendency (e.g. means) or other basic estimates (e.g. regression coefficient) AND variation (e.g. standard deviation) or associated estimates of uncertainty (e.g. confidence intervals)
☐	☒	For null hypothesis testing, the test statistic (e.g. *F*, *t*, *r*) with confidence intervals, effect sizes, degrees of freedom and *P* value noted<br>*Give P values as exact values whenever suitable.*
☒	☐	For Bayesian analysis, information on the choice of priors and Markov chain Monte Carlo settings
☐	☒	For hierarchical and complex designs, identification of the appropriate level for tests and full reporting of outcomes
☐	☒	Estimates of effect sizes (e.g. Cohen's *d*, Pearson's *r*), indicating how they were calculated

Our web collection on statistics for biologists contains articles on many of the points above.

Software and code

Policy information about availability of computer code

Data collection	TCS was performed using a HiSeq 2500 (Illumina).<br>LC-MS/MS; Orbitrap Exploris 480 was used for non-target proteomics.<br>NextSeq500 sequencer was used for RNA sequence.
Data analysis	Graph plotting: GraphPad Prism® Ver 9.4.1<br>Statistical analysis: R ver 3.6.3<br>Mutation calling was performed using Mutect2, VarSCan2, and LoFreq.<br>The pathogenicity of the variants was initially screened using COSMIC (https://cancer.sanger.ac.uk/cosmic). COSMIC-negative variants were then assessed with Mutation Taster (http://www.mutationtaster.org/), PolyPhen2 (http://genetics.bwh.harvard.edu/pph2/dbsearch.shtml) and fathmmMKL (http://fathmm.biocompute.org.uk/fathmmMKL.htm#download).<br>Genome-wide CNAs were analyzed by Python 2.6.6 using CNVkit library version 0.9.1<br>MS: Scaffold DIA (Proteome Software)<br>RNA sequencing: The default parameters of TopHat2 version 2.0.8 and Bowtie2 version 2.1.0 were used to map sequence reads to the human genome (NCBI version 19), and gene annotation information was provided by NCBI. The Cufflinks software tool was used to estimate text abundance (version 2.1.1).

For manuscripts utilizing custom algorithms or software that are central to the research but not yet described in published literature, software must be made available to editors and reviewers. We strongly encourage code deposition in a community repository (e.g. GitHub). See the Nature Portfolio guidelines for submitting code & software for further information.

Data

Policy information about availability of data

All manuscripts must include a data availability statement. This statement should provide the following information, where applicable:

- Accession codes, unique identifiers, or web links for publicly available datasets
- A description of any restrictions on data availability
- For clinical datasets or third party data, please ensure that the statement adheres to our policy

RNA-seq and nontargeted proteomics dataset will be deposited in the GSE213527 and PXD036604. The source data underlying graphs shown in this study are provided in Supplementary Data 6.

Human research participants

Policy information about studies involving human research participants and Sex and Gender in Research.

Reporting on sex and gender	In this report, because we have only analyzed differences in biological attribute, all terms are "sex" and the term "gender" is not used.
Population characteristics	Japanese patients diagnosed clinically GH-producing pituitary adenoma and diagnosed clinically nonfunctional pituitary adenoma were included in this study.
Recruitment	We retrospectively studied patients who underwent transsphenoidal surgery for sporadic acromegaly at Toranomon Hospital between 2013 and 2019, and also studied patients who were diagnosed with NFPAs for comparison. The acromegaly diagnosis was based on typical symptoms, such as a characteristic appearance and the enlargement of the limbs and tongue, in addition to laboratory findings, including elevated basal growth hormone, sex- and age-adjusted IGF-1, and unsuppressed growth hormone after an oral glucose tolerance test, with MRI evidence of pituitary adenoma. Growth hormone immunoreactivity was confirmed histologically in all samples by a pathologist.
Ethics oversight	The collection of adenoma samples and patient information was approved by the ethics committee of Chiba University Graduate School of Medicine and Toranomon Hospital.

Note that full information on the approval of the study protocol must also be provided in the manuscript.

Field-specific reporting

Please select the one below that is the best fit for your research. If you are not sure, read the appropriate sections before making your selection.

☒ Life sciences ☐ Behavioural & social sciences ☐ Ecological, evolutionary & environmental sciences

For a reference copy of the document with all sections, see nature.com/documents/nr-reporting-summary-flat.pdf

Life sciences study design

All studies must disclose on these points even when the disclosure is negative.

Sample size	No sample size determination was performed. Sample sizes were determined to be more than 100 samples based on similar reports on genomic classification of pituitary endocrine tumor (Neou M, et al. Pangenomic Classification of Pituitary Neuroendocrine Tumors. Cancer Cell 37, 123-134 e125, 2020), and proteogenomic analysis (Zhang B, et al. Proteogenomic characterization of human colon and rectal cancer. Nature 513, 382-387, 2014).
Data exclusions	No data were excluded from the analysis.
Replication	Because unique human pituitary adenoma samples were used in this study and no cell lines, primary culture or research animals, replication was not possible.
Randomization	Because patient grouping was based on clinical characteristics and genotype, no randomization was performed.
Blinding	All samples were coded. IHC scorings of SIGMAR1, ATP2A2, ARID5B, WWC3 and SERINC1, counting immunoreactive cells, were performed by two researchers independently.

Reporting for specific materials, systems and methods

We require information from authors about some types of materials, experimental systems and methods used in many studies. Here, indicate whether each material, system or method listed is relevant to your study. If you are not sure if a list item applies to your research, read the appropriate section before selecting a response.

Materials & experimental systems

n/a	Involved in the study	
☐	☒	Antibodies
☒	☐	Eukaryotic cell lines
☒	☐	Palaeontology and archaeology
☒	☐	Animals and other organisms
☐	☒	Clinical data
☒	☐	Dual use research of concern

Methods

n/a	Involved in the study	
☒	☐	ChIP-seq
☒	☐	Flow cytometry
☒	☐	MRI-based neuroimaging

Antibodies

Antibodies used

GH (Dako, A0570, 1:10)
cytokeratin; CK, CAM 5.2 (BD Biosciences, 345779, 1:10)
SSTR2a (Abcam, ab134152, 1:1000)
SIGMAR1 (Sigma-Aldrich, HPA018002, 1:00)
ATP2A2 (Sigma-Aldrich, HPA062605, 1:300)
ARID5B (Sigma-Aldrich, HPA015037, 1:100)
WWC3 (Sigma-Aldrich, HPA039814, 1:500)
SERINC1 (Sigma-Aldrich, HPA035738, 1:50)

Validation

All commercial antibodies were validated as stated in the manufacturer's data sheets.

Clinical data

Policy information about clinical studies

All manuscripts should comply with the ICMJE guidelines for publication of clinical research and a completed CONSORT checklist must be included with all submissions.

Clinical trial registration

The collection of adenoma samples and patient information was approved by the ethics committee of Chiba University Graduate School of Medicine and Toranomon Hospital.

Study protocol

We studied 121 patients who underwent TSS for sporadic acromegaly at Toranomon Hospital between 2013 and 2019, and also studied 46 patients who were diagnosed with NFPAs. The acromegaly diagnosis was based on typical symptoms, such as a characteristic appearance and the enlargement of the limbs and tongue, in addition to laboratory findings, including elevated basal GH, sex- and age-adjusted IGF-1, and unsuppressed GH after an oral glucose tolerance test (OGTT), with MRI evidence of pituitary adenoma. GH immunoreactivity was confirmed histologically in all samples by a pathologist.

Data collection

Data were retrospectively collected from medical records. Patients were recruited until 2019, and data were collected for at least 1 year postoperatively to assess the presence or absence of additional postoperative therapy.

Outcomes

Because this study is not a prospective study, no outocome was set.
